# Unilateral Hemiparesis with Thoracic Epidural in an Adolescent

**DOI:** 10.1155/2012/732584

**Published:** 2012-01-30

**Authors:** Rosalie F. Tassone, Christian Seefelder, Navil F. Sethna

**Affiliations:** ^1^Department of Anesthesiology, Perioperative and Pain Medicine, Children's Hospital Boston, Boston, MA 02115, USA; ^2^Division of Pediatric Anesthesiology, Department of Anesthesiology, University of Illinois Medical Center, 1740 West Taylor Street, Chicago, IL 60612, USA

## Abstract

*Objective*. Unilateral sensory and motor blockade is known to occur with epidural anesthesia but is rarely reported in children. The differential diagnosis should include the presence of a midline epidural septum. *Case Report*. We describe a case of a 16-year-old adolescent who developed repeated complete unilateral extensive epidural sensory and motor blockade with Horner's syndrome after thoracic epidural catheter placement. This unusual presentation of complete hemibody neural blockade has not been reported in the pediatric population. Maneuvers to improve contralateral uniform neural blockade were unsuccessful. An epidurogram was performed to ascertain the correct location of the catheter within the epidural space and presence of sagittal compartmentalization. *Conclusion*. This case report highlights a less frequently reported reason for unilateral sensory and motor blockade with epidural anesthesia in children. The presence of a midline epidural septum should be considered in the differential diagnosis of unilateral epidural blockade.

## 1. Introduction

Unilateral blockade is a known occurrence of epidural analgesia. Frequently, it occurs as a result of patient positioning, lateral displacement of the epidural catheter, or an uneven distribution of the local anesthetic. Additionally, a midline epidural septum may result in unilateral epidural blockade. We report a case of recurrent unilateral thoracic epidural analgesia associated with motor and sensory blockade of one side of the body in an adolescent due to midline epidural septum confirmed by epidurogram. Epidural anatomy, diagnosis, and implication for postoperative analgesia are discussed.

## 2. Case Report

A 16-year-old female, who was 157 cm tall and weighed 54 kg, had a longstanding history of Crohn's disease and presented for an exploratory laparotomy and resection of a small bowel anastomotic stricture. Medications included 6-mercaptopurine, omeprazole, infliximab, lactulose, iron supplements, and mesalamine. The patient and her mother requested epidural analgesia for postoperative pain control. Epidural analgesia had been used with a previous ileocecectomy surgery, and the patient indicated satisfactory postoperative analgesia with apparent unilateral epidural blockade.

After intravenous sedation, the patient was positioned in the left lateral decubitus position and the epidural space was entered using an 18-gauge Tuohy needle and loss of resistance to normal saline at the T9-10 interspace in a midline approach. After negative aspiration for blood and cerebrospinal fluid, a 20-gauge multiorifice epidural catheter was advanced approximately 4 cm in the cephalad direction easily and uneventfully. A test dose of 3 mL of lidocaine 1% with epinephrine 1 : 200,000 was injected without evidence of intravascular or subarachnoid injection.

The patient was then placed in the supine position, and general anesthesia was induced with propofol and vecuronium. The trachea was intubated and anesthesia was maintained with air, oxygen, and isoflurane. Her general anesthetic course was unremarkable.

The epidural catheter was injected with 100 mcg of clonidine and 15 mL of 0.25% bupivacaine. This was followed by an infusion of 0.1% bupivacaine with hydromorphone 10 mcg/mL at 15 mL/hr and was maintained throughout the case.

At the conclusion of surgery, the trachea was extubated and the patient appeared comfortable. In the postanesthesia care unit, it was noted that she had mild left unilateral Horner's sign and weakness of both lower extremities and the epidural infusion rate was decreased to 9 mL/h. She was discharged from the PACU to the ward stable and comfortable.

**Figure 1:**
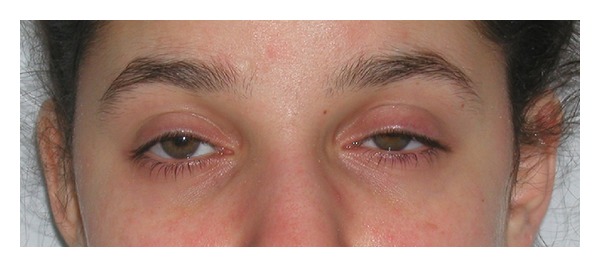
Horner's sign.

**Figure 2:**
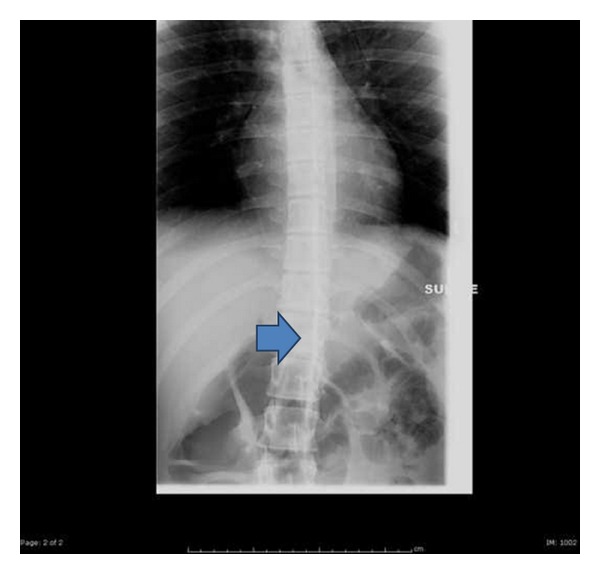
Epidurogram revealing unilateral spread of contrast medium.

On postoperative day one, the patient was comfortable but complained of left sided numbness and visual blur. Physical examination revealed left hemibody analgesia, weakness, and Horner's syndrome (Figure 1). The unilateral dilated pupil was sluggishly reactive to light. Muscle strength was weaker in the upper extremity (3/5) than the lower extremity (4/5) with intense brachial plexus weakness. The epidural rate was further reduced to 8 mL/hr. In view of satisfactory analgesia, the patient and her mother elected to continue with epidural analgesia. The patient was turned to the right lateral decubitus position for several hours to determine if the local anesthetic would gravitate to the contralateral side but the sensory and motor examination remained unchanged. The reduction of epidural infusion produced progressive improvement of numbness, weakness, and the Horner's syndrome over the following 48 hours without compromising analgesia. In light of the history of unilateral epidural block with a previous surgery two years earlier, permission was obtained from the patient and her mother to perform an epidurogram. Two milliliters of contrast medium, iohexol180, was injected while observing anteroposterior fluoroscopic views of the thoracolumbar spine. These views showed left unilateral spread of the contrast medium (Figure 2). Contrast medium spread extended cephalad from T10 to C7, suggesting a midline barrier dividing the epidural space. The epidural catheter was removed on the fourth postoperative day and the neurological signs and symptoms and Horner's syndrome completely resolved. 

## 3. Discussion

We describe a case of an adolescent patient with unilateral epidural blockade and radiographic suggestion of a midline epidural septum. The incidence of unilateral epidural blockade in children and adolescents is unknown. In review of the literature, we found only one case report of an incidental finding of an incomplete posterior midline epidural septum. This was noted in a 5.5-month-old infant when an epidurogram was performed to confirm thoracic placement of an epidural catheter that was advanced from the lumbar region [1, 2].

Epidural posterior midline septum (plica dorsalis medianalis), congenital trabeculation or acquired adhesions have been described in the adult literature but notably are rare [3–5]. It is most frequently noted in the obstetric anesthesia literature as an incidental finding when epidural anesthesia is asymmetrical, despite multiple maneuvers to correct the asymmetry [6]. Although the presence of an epidural posterior and anterior midline septum is proposed to be a diffusion barrier to local anesthetic circumferential spread in the epidural space, the barrier could be incomplete fibrous tissue [1, 5, 7] or fatty tissue [5]. Rarely, a complete impervious epidural midline posterior septum would produce a mechanical barrier that would limit local anesthetic distribution unilaterally leading to ipsilateral anesthesia such as the case in our patient and described in an adult case report [8].

Although rare [5], the existence of an epidural posterior midline connective tissue septum between the dura mater and the ligamentum flavum has been confirmed by radiographic studies and seen frequently during lumbar disc surgery [2], epiduroscopic visualization [9], and CT-epidurography [10].

Another possible cause of unilateral anesthetic blockade in this case could have been subdural placement of the epidural catheter. However, the unilateral anesthetic blockade in our patient was not associated with clinical manifestation of intracranial spread of the local anesthetic such as mental status change, visual and/or bulbar function disturbances due to paralysis of the cranial nerves, nonreactive fully dilated pupil and symmetrical sensory and motor impairments [11]. In addition, the epidurogram in this case was consistent with unilateral epidural spread of the contrast medium rather than the characteristic bilateral pattern reported with subdural spread [7, 11, 12].

The incidence of asymmetrical epidural analgesia in children is unknown. In an earlier report of 202 adults who received a lumbar epidural catheter, the incidence of unilateral epidural analgesia was 5.9%. Replacement of the epidural catheter resulted in bilateral epidural analgesia in all patients [4]. The investigators speculated that the initial catheter was misplaced either in the anterior epidural space or in the paravertebral space via vertebral foramina. The same investigators subsequently examined unilateral epidural analgesia with roentgenographs in a relatively large group of adults who received lumbar epidural catheters and found that in approximately 1.6% of patients the catheter was misplaced in the anterior epidural space and in 1.2% of patients the catheter migrated outside the epidural space via vertebral foramina into the paravertebral space [3]. The use of a therapeutic volume of local anesthetic yielded bilateral epidural analgesia in the group in whom the catheters were within the epidural space. Unilateral epidural analgesia is frequently attributed to catheter placement that is too far anterolateral in the epidural space, nonuniform spread of local anesthetic particularly when small local anesthetic volumes are injected slowly, pooling of local anesthetics in the dependent position, and less frequently to the presence of an epidural midline septum [2]. Migration of the epidural catheter to the paravertebral space may yield paravertebral nerve blockade simulating apparent unilateral epidural analgesia, and the misplacement could be confirmed by obtaining a roentgenograph after injection of contrast medium through the catheter [3, 8, 13].

In summary, we report a case of extensive unilateral epidural sensory and motor blockade most probably due to the presence of a midline posterior and/or anterior septum with adequate postoperative analgesia presumably from eventual spread of the local anesthetics to dural cuffs (root sleeves) and subsequent entry into the cerebral spinal fluid by way of arachnoid granulations [14].
